# The Association of Work Stress and Glycemic Status Is Partially Mediated by Autonomic Nervous System Function: Cross-Sectional Results from the Mannheim Industrial Cohort Study (MICS)

**DOI:** 10.1371/journal.pone.0160743

**Published:** 2016-08-17

**Authors:** Marc N. Jarczok, Julian Koenig, Jian Li, Daniel Mauss, Kristina Hoffmann, Burkhard Schmidt, Joachim E. Fischer, Julian F. Thayer

**Affiliations:** 1 Mannheim Institute of Public Health, Social and Preventive Medicine, Mannheim Medical Faculty, Heidelberg University, Ludolf–Krehl–Strasse 7–11, 68167, Mannheim, Germany; 2 Institute of Medical Psychology, Center for Psychosocial Medicine, UniversityHospital Heidelberg, Heidelberg, Germany, Bergheimer Strasse 20, 69115, Heidelberg, Germany; 3 The Ohio State University, Department of Psychology, 1835 Neil Avenue, 43210, Columbus, Ohio, United States of America; 4 Institute of Occupational, Social, and Environmental Medicine, Center for Health and Society, Faculty of Medicine, University of Düsseldorf, Universitätsstrasse 1, 40225 Düsseldorf, Germany; 5 Department of Occupational Medicine Allianz SE, Königinstrasse 28 80802, Munich, Germany; University of Texas Health Science Center at San Antonio, UNITED STATES

## Abstract

**Background:**

Work stress is associated with an increased risk of pre-diabetes, Type 2 diabetes, and inflammation, as well as decreased autonomic nervous system function as measured, for example, via heart rate variability. We investigated the extent to which the association between work stress and glycemic status is mediated by vagally-mediated heart rate variability (vmHRV) and/or inflammation.

**Methods:**

Cross-sectional data from the Mannheim Industrial Cohort Study (MICS) with 9,937 participants were analyzed. The root mean squared successive differences (RMSSD) from long-term heart rate monitoring during work and night time periods was used to index vmHRV. Fasting plasma glucose and glycosylated hemoglobin were assessed to determine glycemic status. High sensitive C-reactive protein levels were observed as a measure of systemic inflammation and the Effort-Reward-Imbalance scale was used to evaluate work stress. Mediation models were adjusted for age, sex, and occupational status, and estimations were bootstrapped (5,000 replications).

**Results:**

Effort-Reward-Imbalance was significantly negatively associated with RMSSD and both glycosylated hemoglobin and fasting plasma glucose during both work and night time periods. Effort-Reward-Imbalance was observed to have a significant direct effect on glycosylated hemoglobin and significant indirect effects, through RMSSD, on both glycemic measures during both time periods. Introducing C-reactive protein as a further mediator to the model did not alter the indirect effects observed. C-reactive protein, as an exclusive mediator, was observed to have smaller direct and indirect effects on the glycemic measures as compared to when Effort-Reward-Imbalance was included in the model.

**Conclusions:**

Our results suggest that the association between work stress and glycemic status is partially mediated through vmHRV independent of systemic inflammation as measured by C-reactive protein. We conclude that work stress may be an additional factor that promotes development of hyperglycemic-metabolic states. If supported by prospective evidence, these results may lead to new approaches for primary prevention of hyperglycemia in the workplace.

## Introduction

The World Health Organization (WHO) declared work-related stress as one of the primary challenges in the 21^st^ century [1]. Work stress, described as adverse psychosocial work characteristics, is broadly recognized as a significant determinant for health [2,3], associated with the development of cardiovascular disease (CVD) [3,4], the pathogenesis of essential hypertension [5], the onset of Metabolic Syndrome (MetS) [6,7], and increased HbA_1c_ levels [8,9]. MetS and CVD (including essential hypertension) are major causes of morbidity and mortality globally for men and women across all age groups [10]. Stress is thought to exert negative effects on health through constant stimulation of a number of physiologic systems, including the hypothalamic-pituitary-adrenal axis, the immune system, and the autonomic nervous system (ANS). Although MetS has consistently been associated with work stress [6,7], the mechanisms remain poorly understood [6]. A core element of the definition of MetS is the presence of hyperglycemic status; whereas cut-off values for other elements, such as hypertension, indicators for dyslipidemia, and abdominal adiposity, vary.

As we have previously shown, ANS function and glycemic status are significantly associated with each other in healthy working adults, independent of other components of MetS [11] and levels of cortisol, C-reactive protein (CRP, a marker of systemic inflammation), and norepinephrine (a marker for sympathetic nervous system activity) [12]. This suggests a prominent role for glycemic status in the etiology of MetS and downstream-related diseases such as CVD. In addition, we reported work stress to be significantly positively correlated with glycemic status before, but not after, ANS function is entered into adjusted models. This finding suggests a potential mediating role of ANS function between work stress and glycemic regulation [12].

Psychosocial stress, including work stress, has been shown by ourselves and others to be associated with an increased risk of pre-diabetes and Type 2 diabetes, but the underlying mechanism is not fully elucidated [13–18]. An increased perception of work stress is also known to be negatively associated with ANS function [19].

One widely used measure of ANS function is the beat-to-beat variation in cardiac rhythm, as indexed by heart rate variability (HRV). Many organs in the human body are dually controlled by sympathetic and parasympathetic nerves, contributing to modulation of energy expenditure. For example, indices of cardiac function such as heart rate (HR) are determined by inherent cardiac mechanisms and the joint activity of the sympathetic nerve and vagus (the primary parasympathetic) nerve at the sinoatrial node. Here, relative increases in parasympathetic activity (vs. sympathetic) have positive (vs. negative) chronotropic effect [20]. The differential effects of the ANS on the sinoatrial node, and thus the timing of the heart beats, are due to the differential effects of the neurotransmitters for the sympathetic (norepinephrine) and parasympathetic (acetylcholine) nervous systems. Usually, both branches of the ANS are tonically active (sympatho-vagal balance), but their opposing effects are not algebraically additive. The heart is known to be under tonic inhibitory control by parasympathetic means (i.e. vagus nerve), as the normal resting heart rate is about 30 BPM lower compared to intrinsic heart rate when both, sympathetic and parasympathetic influences are blocked pharmacologically (double blockade [21]). Therefore, energy conservation is favored at rest by parasympathetic dominance over sympathetic influence [22]. Moreover, sympathetic effects on the heart are slow, on the time scale of seconds, whereas the parasympathetic effects are fast, on the time scale of milliseconds. The beat to beat series is usually characterized by high variability in heart rate supporting the notion of vagal dominance as sympathetic influence at the heart is to slow to generate rapid changes because the parasympathetic influences are the only ones capable of producing rapid changes in the beat to beat timing of the heart [23]. Certain HRV measures specifically capture rapid changes in HR, such as the square root of the mean of the sum of the squares of differences between adjacent NN intervals. As such, RMSSD is considered an index of primarily parasympathetic control. The vagus nerve has been known for some time to play a significant role in health and disease. For example, Charles Darwin cited Claude Bernand’s earlier work on the role of the pneumogastric (i.e. vagus) nerve under excitement [24]. Vagus nerve activity is central in systemic down modulation of inflammation, coined the „cholinergic anti-inflammatory pathway”[25,26], with subclinical inflammation previously associated with both hyperglycemia and whole-body insulin resistance in Type 2 diabetes [27].

HRV may be more than just an index of healthy heart function. HRV may, in fact, serve as an easy output measure of the brain’s integrative systems responsible for adaptive regulation [22]. Measures of HRV are independently associated with work stress and the morbidity and mortality from a wide range of disorders including inflammation, fatigue, and CVD [3,28,29].

Lane proposed four levels of functioning that should be addressed in psychosomatic research, investigating pathways from mind to body and vice versa. These levels are (1) mental/psychological/behavioral states and traits; (2) the brain (so called “wet ware”); (3) information transfer systems (i.e., the autonomic nervous system, endocrine system, and immune system); and (4) the body proper (i.e., end organ function and dysfunction) ([30] p.118). The neurovisceral integration model is one theory of how the perception of stress (level 1) is processed by the brain (level 2) and through information transfer systems (level 3) to impact end organ (dys)function (level 4) [22,31,32]. The neurovisceral integration model specifically integrates autonomic, attentional, behavioral, and affective systems into a functional and structural network [31]. For example, successful adaptation to environmental challenges requires input from many sources including physiological, behavioral, affective, cognitive, social, and environmental sources. Two neural structures involved in perceptions of threat and safety are pivotal to the neurovisceral integration model: the amygdala and the medial prefrontal cortex (mPFC) [22]. The latter has a significant role in the representation of internal and external context in the brain and has been linked to self-judgments of health [33]. Both internal and external contextual information is used to regulate behavior and peripheral physiology. In particular, the ventromedial PFC is specifically involved in a) building the meaning of a situation, especially when conceptual information drives affective, physiological, and behavioral responses [34] and b) the regulation of the fear/threat response [22,35]. Furthermore, frontal cortex structures are particularly involved in visceral sensory processing with an emphasis on the medial prefrontal region [36].

The model of neurovisceral integration proposes that the “default” response to uncertainty, novelty, and threat is “the sympathoexcitatory preparation for action commonly known as the fight or flight response” [22]. A continued perception of threat or stress, however, is maladaptive and well known to be a primary factor for general health decline; for example, as captured by the allostatic load model [37,38] and specifically for psychosocial factors such as work stress [19,39,40]. Thus, there is a need to appropriately down regulate the default response to uncertainty, novelty, and threat in safe environments. This down regulation occurs in the ventromedial PFC region. The amygdala is tonically inhibited by an active vmPFC region, while the threat response is disinhibited (rather that activated) by a hypoactive vmPFC in situations of uncertainty and threat [22,31]. This view leads us to ask, “Why is the stress system not inhibited?” as compared to the question asked in classical stress theories, “Why is the stress system active?”, shifting the focus from sympathoexcitatory activation such as cortisol outflow to the inhibitory role of the vmPFC region and the connectivity especially between the vmPFC and amygdala. A peripheral physiological, noninvasive proxy measure of the vmPFC region activity can be inferred from vagally mediated measures of heart rate variability at the sinoaterial node of the heart (Please see [31] for a complete description on the neural pathways between vmPFC and the sinoatrial node of the heart).

The objective of our study was to investigate if, and to what extent, the association between work stress and glycemic status (as indicated by both glycosylated hemoglobin and fasting plasma glucose) is mediated through 1) autonomic nervous system function as indexed by heart rate variability and 2) inflammation as indexed via high sensitive C-reactive protein.

## Material and Methods

### Study Sample

We utilized a large cross-sectional, secondary data set from the Mannheim Industrial Cohort Study (MICS). The data were collected as part of a voluntary health risk assessment that was offered to all employees during working hours. An agent independent from the employer conducted the health risk assessments and data collection (HealthVision Ltd., Berlingen, Switzerland). Data on 9,924 participants was collected from 13 study sites (companies from the secondary and tertiary sectors) in Germany between 2010 and 2012. The overall participation rate was 55% (range 49%-60%). Participants were invited to take part in a “Work Health Check” and were offered a detailed individual report of their health status as assessed by physical examination and self-reports. The study population encompassed the entire workforce at the 13 sites between 18 and 65 years of age. The Ethical Committee of the Mannheim Medical Faculty, Heidelberg University approved the secondary analysis study (2010-296E-MA). All participants gave written informed consent prior to the “Work Health Check”.

### Measures

Demographic, medical, and lifestyle variables were obtained from an online questionnaire. The questionnaire was completed prior to scheduling the physical examination. All participants were enrolled and examined between 10 a.m. and 5 p.m. on a typical workday (Monday to Friday).

Work stress was assessed using the Effort-Reward Imbalance model (ERI). The ERI model is based on social reciprocity, a core principle of social action. Applied to the work context, people trade their labor against payment, appraisal, and career advancement. According to the ERI model, stress at work exists if an imbalance appears in work input (the effort scale) and gratification (the reward scale). Reciprocity is violated when the ratio of effort/reward becomes larger than one. High efforts and low rewards are associated with higher stress at work. Failed reciprocity can be defined either as a binary variable (i.e., if an imbalance between high ‘‘cost” and low ‘‘gain” exists / does not exist) or continuously. In a prior systematic study, the use of a continuous variable was shown to be a better predictor of health as compared to the use of a binary outcome [41]. Details on the psychometric properties of the ERI model are published elsewhere [42].

HR was recorded as beat-to-beat intervals (IBI) using a t6 Suunto Memory Belt (SuuntoVantaa, Finland), sampling at a rate of 1000Hz. The Suunto Memory Belt is a reliable measure of electrocardiography (ECG) compared to a 5 lead ECG [43].

IBIs were measured as the interval between two successive R-spikes. After attaching the ambulatory HR recorder, participants continued their routine work duties, followed by after work leisure and sleep activities. Participants were asked to return the HR recorder after a minimum of 22 hours of wearing the device or in case of any difficulties. The next morning, between 7 a.m. and 9 a.m., a fasting blood sample was collected from all participants. Samples were transported to a commercial laboratory (Synlab, Augsburg, Germany) within 2 hours of sample collection and analyzed within 24-hours. Glycemic status and inflammatory status were determined using routine laboratory analysis for fasting plasma glucose [FPG] (OSR6121, Olympus), glycosylated hemoglobin [HbA_1c_] (“Tina-quant, Hemoglobin A1c Gen.2”, Roche Diagnostics [44]) and high sensitivity C-reactive protein [CRP] (CardioPhase hsCRP, DADE BEHRING). Body mass index (BMI) was assessed and classified according to the WHO standard [45].

RMSSD was calculated from 24 hour long-term HR monitoring (beat to beat) derived from 5.35-minute averages recorded for work time and night time as indicators of parasympathetic control. This index uses what the econometrics literature calls “first-differencing” and acts like a high pass filter, removing long-term trends and slower-frequency variability from the signal. RMSSD primarily reflects vagal influences [46] because of the frequency characteristics of the autonomic influences on the heart, specifically that vagal influences cover the full frequency range and sympathetic influences are primarily restricted to the lower frequencies [47].

Researchers at the Center for Neuropsychological Research (University of Trier, Germany) analyzed the raw IBIs according to the “Task Force Guidelines of the European Society of Cardiology and the North American Society of Pacing and Electrophysiology”. The “ANS-Explorer” Software [48] was used to calculate the RMSSD in all valid adjacent IBIs if the artifact rate of the according 5.35 minute segment was below 5%. Due to the skewness of the data, the HRV and CRP measures were log-transformed according to the ladder of power [49].

The occupational status (blue vs. white collar) was determined via the online questionnaire using the question, “What best describes your current position?”; 1) Division manager / Department manager: 2) Project leader, process manager, team leader, foreman, group leader; 3) Employee; 4) Skilled worker; 5) unskilled worker). Occupational status was also based on the self-reported main activity / occupation at work (e.g., “Produce or finish components with tools” (indicator for blue collar) or “Administration, secretarial work” (indicator for white collar)). The characteristics of study subjects are reported in Table 1.

**Table 1:** Characteristics of study subjects N = 9,937.

Variable	Mean or %	SD
ERI	1.2	0.5
ERI >1	71.1%	
Female	18.2%	
Age (Years)	41.2	10.6
RMSSD night (msec)	41.2	22.0
RMSSD day (msec)	29.1	13.6
HbA_1c_ (% [mmol/mol])	5.5 [3.7]	0.5
<6% (<42 mmol/mol)	92%	
6–<6.5% (42–<48 mmol/mol)	5.9%	
≥6.5% (≥48 mmol/mol)	2.1%	
FPG (mg/dl)	86.8	13.6
< 80 mg/dl	22.5%	
80–<100 mg/dl	69.4%	
100–<126 mg/dl	6.8%	
≥126 mg/dl	1.3%	
Blue Collar	67.3%	
BMI (kg/m^2^)	26.0	4.2
Underweight (<18.5)	1.1%	
Normal weight (18.5–<25)	44.3%	
Overweight (25–<30)	40.2%	
Obese (>30)	14.5%	
High sensitive CRP	1.71	3.09
Low (<1.0 mg/L)	53.5%	
Medium (1.0 –<3.0 mg/L)	32.6%	
High (>3.0 mg/L)	13.9%	

### Statistical Analysis

We used STATA 12.1 MP (College Station, TX) for all analyses. We estimated four distinct mediation models (MM) using a structural equation approach as suggested by Ditlevsen et al. [50]. One of the main advantages of this approach is a simultaneous estimation of all specified pathways.

1. MM1 investigated the effect of work stress (independent variable X) on glycemic status (dependent variable Y) via ANS function (mediator variable M_1_) and inflammation (mediator variable M_2_) simultaneously.
2. MM2 investigated the effect of work stress (independent variable X) on glycemic status (dependent variable Y) via ANS function (mediator variable M_1_).
3. MM3 investigated the effect of work stress (independent variable X) on glycemic status (dependent variable Y) via inflammation (mediator variable M_2_).
4. MM4 investigated the effect of inflammation (independent variable X) on glycemic status (dependent variable Y) via ANS function (mediator variable M_1_).

**Fig 1:**
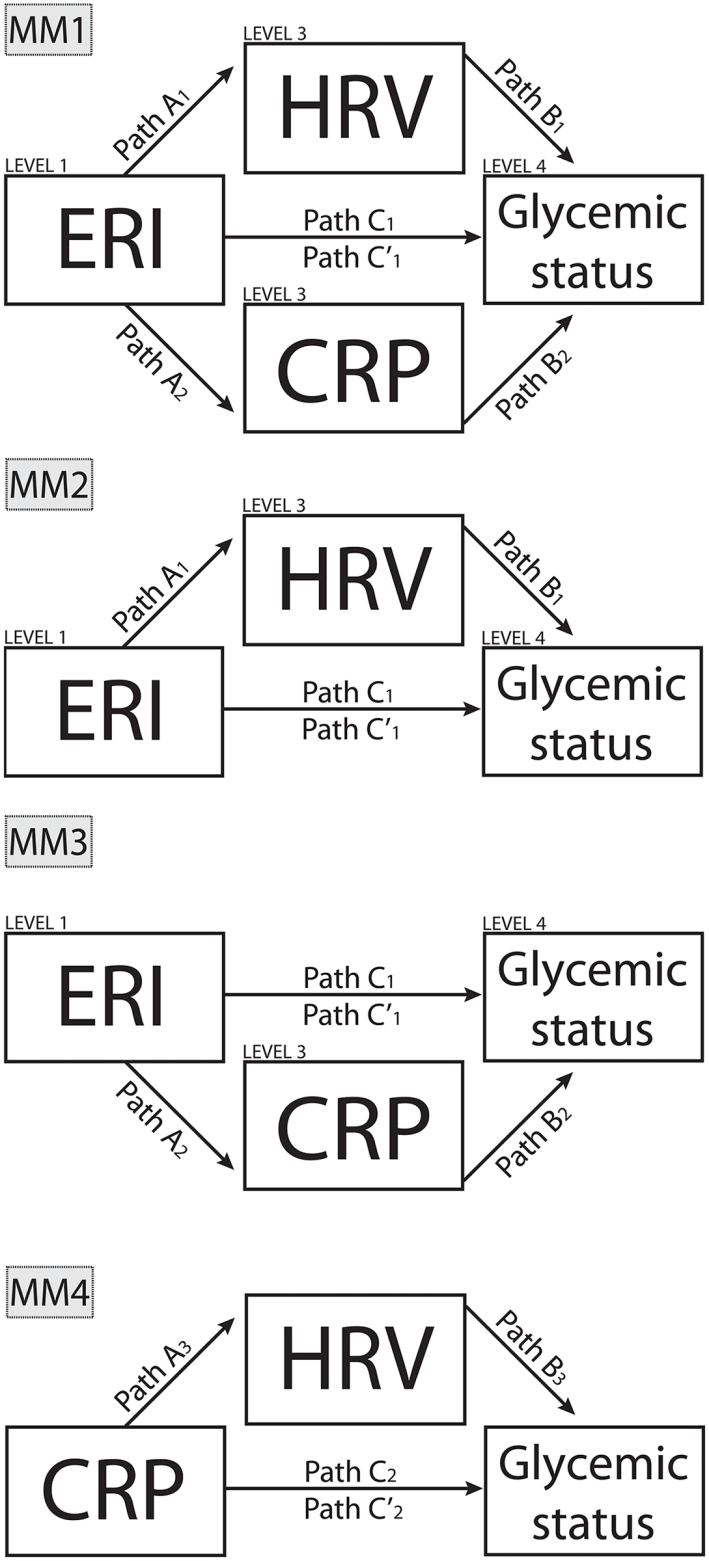
Conceptual mediation models (MM). MM1 represents the full conceptual model, MM2 and MM3 represent nested models. MM4 is testing an alternative causal relationship.

MM2 and MM3 were nested models of MM1 that assumed the mediation of CRP (in MM2) or ANS function (MM3) to be zero. We operationalized ERI as X, RMSSD measured during the day or night as M_1,_ CRP as M_2_, and FPG or HbA_1c_ as Y in MM1 through MM3. In MM4 we operationalized CRP as X, RMSSD measured during the day or night as M_1,_ and FPG or HbA_1c_ as Y (as depicted in Fig 1).

Following the procedure for mediation analysis suggested by Hayes et al. (2009) [51], six equations were identified to estimate the direct, indirect, and total effects in MM1, and four equations in MM2, MM3, and MM4. The proportion mediated was also calculated (see Appendix A Eq A7). All models were adjusted for the potential confounders of age (C_1_), sex (C_2_), and occupational status (blue vs. white collar) (C_3_). The indirect and total effects are reported within Table 2, the direct effect is equivalent to the total effect of [Path C´, see Appendix A].

**Table 2:** Unstandardized regression coefficient for mediation models 1 to 4.

Path	FPG & HRV Day			FPG & HRV Night			HbA1c & HRV Day			HbA1c & HRV Night		
**Model 1**	**Coeff**	**SE**	**z**^§^	**Coeff**	**SE**	**z**^§^	**Coeff**	**SE**	**z**^§^	**Coeff**	**SE**	**z**^§^
(A_1_) X→M_1_	**-0.02**	**0.01**	**-2.49**	**-0.03**	**0.01**	**-2.82**	**-0.02**	**0.01**	**-2.57**	**-0.03**	**0.01**	**-2.85**
(A_2_) X→M_2_	**0.12**	**0.03**	**3.62**	**0.12**	**0.03**	**3.62**	**0.12**	**0.03**	**3.62**	**0.12**	**0.03**	**3.62**
(B_1_) M_1_→Y	**-4.53**	**0.47**	**-9.68**	**-1.98**	**0.38**	**-5.15**	**-0.13**	**0.02**	**-7.29**	**-0.06**	**0.01**	**-4.44**
(B_2_) M_2_→Y	0.10	0.08	1.29	**0.19**	**0.08**	**2.34**	0.00	0.00	-1.26	0.00	0.00	-0.45
(C_1_) X→Y	0.42	0.30	1.37	0.42	0.30	1.37	**0.03**	**0.01**	**2.61**	**0.03**	**0.01**	**2.61**
(C'_1_) X→Y	0.30	0.30	0.98	0.33	0.31	1.07	**0.03**	**0.01**	**2.37**	**0.03**	**0.01**	**2.44**
*Indirect effect*	**0.12**	**0.05**	**2.63**	**0.09**	**0.03**	**3.13**	**0.00**	**0.00**	**2.07**	**0.00**	**0.00**	**2.09**
*Total effect*	0.42	0.30	1.38	0.41	0.30	1.37	**0.03**	**0.01**	**2.61**	**0.03**	**0.01**	**2.61**
**Model 2**	**Coeff**	**SE**	**z**^§^	**Coeff**	**SE**	**z**^§^	**Coeff**	**SE**	**z**^§^	**Coeff**	**SE**	**z**^§^
(A_1_) X→M_1_	**-0.02**	**0.01**	**-2.49**	**-0.03**	**0.01**	**-2.82**	**-0.02**	**0.01**	**-2.57**	**-0.03**	**0.01**	**-2.85**
(B_1_) M_1_→Y	**-4.59**	**0.47**	**-9.87**	**-1.96**	**0.38**	**-5.1**	**-0.12**	**0.02**	**-7.24**	**0.12**	**0.03**	**3.62**
(C_1_) X→Y	0.44	0.3	1.45	0.44	0.3	1.45	**0.03**	**0.01**	**2.61**	**-0.06**	**0.01**	**-4.44**
(C'_1_) X→Y	0.33	0.3	1.09	0.37	0.31	1.22	**0.03**	**0.01**	**2.34**	0.00	0.00	-0.45
*Indirect effect*	**0.11**	**0.05**	**2.43**	**0.06**	**0.03**	**2.53**	**0.00**	**0.00**	**2.43**	**0.03**	**0.01**	**2.61**
*Total effect*	0.44	0.3	1.45	0.44	0.3	1.44	**0.03**	**0.01**	**2.61**	**0.03**	**0.01**	**2.44**
**Model 4**	**Coeff**	**SE**	**z**^§^	**Coeff**	**SE**	**z**^§^	**Coeff**	**SE**	**z**^§^	**Coeff**	**SE**	**z**^§^
(A_3_) X→M_1_	**-0.02**	**0.00**	**-7.72**	0.00	0.00	0.11	**-0.02**	**0.00**	**-7.17**	0.00	0.00	0.12
(B_3_) M_1_→Y	**-4.54**	**0.47**	**-9.68**	**-1.98**	**0.38**	**-5.15**	**-0.13**	**0.02**	**-7.17**	**-0.06**	**0.01**	**-4.44**
(C_2_) X→Y	**0.19**	**0.08**	**2.32**	**0.19**	**0.08**	**2.32**	0.00	0.00	-0.45	0.00	0.00	-0.45
(C'_2_) X→Y	0.08	0.08	1.04	**0.19**	**0.07**	**2.78**	0.00	0.00	-1.93	0.00	0.00	-0.64
*Indirect effect*	**0.10**	**0.02**	**5.99**	0.00	0.01	-0.11	**0.00**	**0.00**	**5.12**	0.00	0.00	-0.12
*Total effect*	**0.19**	**0.08**	**2.33**	**0.19**	**0.08**	**2.32**	0.00	0.00	-0.66	0.00	0.00	-0.45
**Model 3**#	**Coeff**	**SE**	**z**^§^				**Coeff**	**SE**	**z**^§^			
(A_2_) X→M_2_	**0.12**	**0.03**	**3.62**				**0.12**	**0.03**	**3.62**			
(B_2_) M_2_→Y	**0.19**	**0.08**	**2.32**				0.00	0.00	-0.45			
(C_1_) X→Y	0.44	0.30	1.45				**0.03**	**0.01**	**2.61**			
(C'_1_) X→Y	0.39	0.30	1.30				**0.03**	**0.01**	**2.62**			
*Indirect effect*	**0.02**	**0.01**	**1.96**				0.00	0.00	-0.45			
*Total effect*	0.42	0.30	1.37				**0.03**	**0.01**	**2.61**			

All models were adjusted for age, sex, and occupational status. Coeff = Unstandardized regression coefficient; SE = Standard Error; **Bold** values are significant with p<0.05; N = 9,937

^§^ z-value≥1.96 = p≤0.05; z-value ≥ 2.57 = p≤0.01; z-value ≥ 3.29 = p≤0.001

^#^ Model 3: No HRV measures included in this Model, therefore no day or night measures were reported

The model parameters were estimated with a maximum likelihood procedure allowing for missing values (Expectation-Maximization). We used an alternative robust variance estimation method (bootstrapping with 5000 replications stratified by study site) to additionally adjust for between study site variations. Coefficients were not standardized to keep the original metric of X and Y, allowing meaningful interpretation of the indirect effect. For example, an indirect effect of ERI (X) on HbA_1c_ (Y) can be interpreted as the difference in HbA_1c_ unit (%) attributional to the indirect pathway through the mediators HRV (see reference [51] for an in-depth discussion). The appendix provides further details on specified equations and the STATA commands applied.

## Results

The final sample consisted of a total of 9,924 participants from 13 study sites with an average of 765 participants (SD 661) per site. The mean age was 41.9 years (SD 10.9) and 19% were females (Table 1). One third of the sample was blue collar workers. Across clinical fasting glucose groups, we found significant, clinically relevant differences between groups as indicated by ANOVA (continuous variables) or CHI^2^ (categorical variables) (see Table 3).

**Table 3:** Fasting plasma glucose.

	Supernormal (< 80 mg/dl)		Normal (80–<100 mg/dl)		Prediabetic range (100–<126 mg/dl)		Diabetic range (≥126 mg/dl)		ANOVA or CHI2
	Mean or %	SD	Mean or %	SD	Mean or %	SD	Mean or %	SD	p-value
**ERI**	1.2	0.5	1.2	0.5	1.3	0.5	1.4	0.5	<0.001
**Female (%)**	27%		16%		8%		7%		<0.001
**Age (years)**	36.8	10.5	41.8	10.2	48.3	9.0	50.9	7.4	<0.001
**RMSSD night (msec)**	46.9	24.5	40.3	21.0	32.2	17.6	28.2	19.2	<0.001
**RMSSD day (msec)**	33.5	14.4	28.5	13.0	21.9	10.5	18.6	9.8	<0.001
**HbA**_**1c**_ **(%)**	5.4	0.4	5.5	0.3	5.8	0.5	7.6	1.7	<0.001
**FPG (mg/dl)**	74.5	5.7	87.4	5.0	106.7	6.5	161.9	43.2	<0.001
**Bluecollar**	65%		68%		56%		59%		<0.001
**BMI (kg/m**^**2**^**)**	24.9	3.9	26.0	4.1	28.8	4.7	30.5	4.9	<0.001
**High sensitive CRP (mg/L)**	1.7	3.1	1.6	3.0	2.3	3.7	2.9	4.2	<0.001
N = 9,937	22.5%		69.4%		6.8%		1.3%		

According to the Methods described above, four distinct models (daytime HbA_1c_, daytime FPG, nighttime HbA_1c_, and nighttime FPG) were calculated per mediation model.

### Mediation Model 1

ERI was significantly negatively associated with RMSSD [Path A_1_] and positively with CRP [Path A_2_, see Appendix A] at day and nighttime for both FPG and HbA_1c_. Furthermore, RMSSD was significantly negatively associated with both FPG and HbA_1c_ during both day and nighttime [Path B_1_]. In contrast, no association was found between CRP and glycemic status, except for FPG in the nighttime model [Path B_2_].

ERI was significantly positively associated with HbA_1c_, but not with FPG in both time periods [Path C_1_] and [Path C’_1_]. Post estimation results indicate significant indirect effects of ERI on glycemic status in all four models. In addition, ERI had a significant direct and total effect during both day and nighttime on HbA_1c_, but not on FPG (Table 2). These results suggest that HRV mediates the relationship between ERI and glycemic status, largely independent of CRP.

### Mediation Model 2

ERI was significantly negatively associated with RMSSD [Path A_1_, see Appendix A] at day and nighttime for both FPG and HbA_1c_. Furthermore, RMSSD was significantly negatively associated with both FPG and HbA_1c_ during both day and nighttime [Path B_1_, see Appendix A]. ERI was significantly positively associated with HbA_1c_, but not with FPG in both time periods [Path C_1_, see Appendix A] and [Path C’_1_]. Post estimation results indicate significant indirect effects of ERI mediated through RMSSD in all four models. In addition, ERI had a significant direct and total effect during both day and nighttime on HbA_1c_, but not on FPG. During daytime, the total effect of ERI on glycemic status appears to be mediated in the daytime models with a proportion of 25.03% (FPG) and 10.15% (87 mmol/mol) (HbA_1c_) and in the night time models of 14.6% (FPG) and 6.7% (50 mmol/mol) (HbA_1c_), respectively (Table 2). The proportion mediated is in a comparable proportion to MM1.

### Mediation Model 3

ERI was significantly positively associated with CRP [Path A_2_] at day and night time for both FPG and HbA_1c_. No association was found between CRP and HbA_1c_, but was found for CRP and FPG [Path B_2_]. Again, ERI was significantly positively associated with HbA_1c_, but not with FPG in both time periods [Path C_1_] and [Path C’_1_] (Table 2).

Post estimation results indicate significant indirect effects of ERI on glycemic status for FPG. In addition, ERI had a significant direct and total effect on HbA_1c_, but not on FPG (Table 2). The proportion mediated is 5.2% for FPG and 0.5% for HbA_1c_.

### Mediation Model 4

CRP was significantly negatively associated with RMSSD [Path A_3_] at day, but not at night time for both FPG and HbA_1c_. Furthermore, RMSSD was significantly negatively associated with both FPG and HbA_1c_ during both day and night times [Path B_3_] (Table 2).

CRP was significantly positively associated with FPG, but not with HbA_1c_ [Path C_2_]. CRP was positively, but not significantly, associated through all HbA_1c_ day and night time models and the FPG daytime model [Path C’_2_]. Post estimation results indicated a significant indirect effect of CRP on FPG, but not on HbA_1c_ (Table 2). These results suggest that part of the effect of CRP on FPG is mediated by HRV (e.g. the proportion mediated is 55% in the daytime model).

## Discussion

In the present paper we investigated to what extent the association between work stress, as indicated by the Effort-Reward Imbalance and glycemic status, is mediated through primarily vagally-mediated HRV, as indicated by RMSSD and systemic inflammation (denoted by CRP during day and during nighttime). We found that the indirect effect of work stress on glycemic status accounts for approximately one quarter of the total effect. We consistently found a significant indirect effect of work stress on glycemic status via vagally mediated HRV.

Part of the inflammation–glycemic status relationship seems to be attributed to ANS function as well. Although there was only one significant association of CRP on FPG, this association appeared to be driven by measures of vagally-mediated HRV and thus parasympathetic (i.e. vagal) activity. Therefore, HRV seems to be a more inclusive measure than CRP. The dual mediation model (MM1) results are similar to the single HRV model´s (MM2) results, suggesting a primarily vagally-mediated HRV driven mediation between ERI and glycemic status. This result is not completely surprising, given the role of vagus nerve activity in inflammation as exemplified by the cholinergic anti-inflammatory reflex and its role in metabolic diseases [28,52].

Subclinical inflammation plays a critical role in the pathogenesis of Type 2 diabetes and insulin resistance syndrome, whereby pro-inflammatory cytokines such as TNF-α levels are increased in obesity and Type 2 diabetes [27]. One of the potential mechanisms of increased subclinical inflammation on an intracellular level encompasses the regulation of TNF-α converting enzyme (TACE) and its inhibitor, tissue inhibitor of metalloproteinase 3 (TIMP3) [53–55]. Here, signal transducer and activator of transcription 3 (STAT3) are modulated by MicroRNAs (miR-124), resulting in a decrease IL-6 production and TACE release [52]. Thus, miR-124 is suggested to mediate the cholinergic anti-inflammatory action via the α7-nicotinic acetylcholine receptor (α7nAChR) through inhibition of the production of pro-inflammatory cytokines [52].

There are several compelling reasons why the work stress—glycemic status relationship appears to be attributable to ANS function. First, it has been consistently shown that work stress is an independent risk factor for the onset of pre-diabetes and Type 2 diabetes [13,15] and increased HbA_1c_ levels [8,9]. Consistent with previous research, we found work stress positively associated with glycemic status measures, a core element in defining MetS. That is, a higher effort reward imbalance (indicating work stress) is associated with an increase in glycemic measures, independent of age, sex, and occupational status. Second, we and others have shown that measures of vagally-mediated HRV have independent associations with glucose regulation [11,12,56]. In line with previous research, we found that vagally mediated HRV is negatively associated with glycemic status. Specifically, higher RMSSD was associated with decreased fasting plasma glucose and decreased glycosylated hemoglobin. And third, it is well known that stress reduces vagally mediated HRV, as recent reviews support [3,19]. We also found that our measure of work stress was significantly and negatively associated with our measure of vagally-mediated HRV.

A wide range of organ systems in the body are innervated via the vagus nerve; as such, the vagus nerve is well positioned to provide feedback about the state of the organism to the brain. Between 50% to 80% of vagal nerve fibers are afferent, supporting the vagus as an important information transfer system, covering immune status, blood glucose levels, and pain [57–59].

The fact, that the ANS plays an important role in immune function is generally accepted today [60,61]. For example, the vagus nerve is known to relay information about the peripheral immune status to the brain via interleukin-1 cytokine receptors conveyed by paraganglia cells situated in parasympathetic ganglia [62]. In addition, efferent vagal activity inhibits the release of pro-inflammatory cytokines via the release of acetylcholine, termed the cholinergic anti-inflammatory pathway [29,63].

Not so well known, however, is the role of the vagus in glucose regulation. Previous research suggests that work stress may increase HbA_1c_ directly via activation of psychoneuroendocrine pathways [60]. Here, the release of catecholamines (e.g. adrenaline, norepinephrine) and glucocorticoids (e.g. cortisol) results in an increased hepatic glucose output. However, we have previously shown that vagal tone seems to play a role in glucose regulation independent of cortisol and norepinephrine [12]. Vagal afferents in the hepatic portal contain glucagon-like peptide-1 receptors (GLP-1r) that convey information about peripheral glucose status to the brain. In addition, efferent vagal outflow to the liver decreases glucose production [64,65]. Thus the action of the vagus nerve is a common denominator for both glucose and immune regulation [28]. In addition, HRV may serve as an important index of this bi-directional communication across diverse bodily systems.

As previously described, Lane et al. [30] distinguished four levels of functioning in the search for pathways from mind to body and vice versa. These are 1) mental/psychological/behavioral states and traits; 2) the brain itself, (e.g., certain circuits); 3) information transfer systems (i.e., ANS and endocrine and immune systems); and 4) the body proper (i.e., end organ function and dysfunction) ([30] p.118). As the authors [30] point out, one needs to cross all four layers for a deeper and more integrative understanding of mind-body interactions and to advance the field of psychosomatic medicine. Within our present work we distinctly showed how a mental state on level 1 (i.e. the perception of work stress) is partly mediated via the ANS independent of immune function (level 3) to glycemic status as indicators of end organ function (level 4). Although we were not able to include level (2) within this work, literature supports a network of neural structures important in the integration and regulation of many systems of the body described as the medial prefrontal-brainstem axis [66]. An important component of this network is the prefrontal cortex. The prefrontal cortex has been linked to immune function as well as glucose regulation. For example, Page et al. [67] reported that higher circulating glucose levels are associated with greater activity in the medial prefrontal cortex. Activity in this area increased in response to glucose infusion and was related to decreased interest in food stimuli. Importantly, this inhibitory control over food motivation was absent in obese individuals. These findings suggest that the prefrontal cortex is an important site for the regulation of glucose and eating behavior, as greater activity in this region is associated with euglycemia and context appropriate eating behavior. Of particular relevance to the present study, we previously reported that HRV is directly related to activity in the medial prefrontal cortex [22].

The Copenhagen City Heart Study [68] reports HbA_1c_ to be significantly associated in a non-diabetic population with incident fatal and nonfatal CVD events in multivariate analyses with a hazard ratio of HR 1.31 (95% CI 1.05–1.64, P = 0.018). In other words, for every unit increase in HbA_1c_, the risk for an incident CVD event was increased by 31%. Under the assumption that these results are transferable, one could estimate the following:

In our study, the work stress measure varied between 0.8 (minimum) and 5 (maximum). Multiplied with the total effect in mediation model 2 of work stress on HbA1c of 0.03 results in an estimated increase in HbA1c between 0.024 (minimum) and 0.15 (maximum). This would indicate in an increase of a potential CVD event of between 0.07% and 4.7%.

### Limitations

There are several possible limitations to consider in the present study. First, the number of women in the sample was relatively small as compared to the general working population. Thus, future research would do well to examine more clearly whether the present findings are generalizable to women. Furthermore, the present sample primarily included individuals of western European descent. As such, our results may not generalize to other ethnicities. Third, as our study was cross-sectional, we cannot draw a cause-effect conclusion. However, there is a growing body of evidence supporting our implied temporal relationship. For example, it has been shown [3,19] that work stress precedes decreases in autonomic activity [Path A_1_]. Also, other studies [64,65] have shown that increasing efferent vagal outflow to the liver decreases glucose production and vice versa [Path B_1_]. And finally, some [16–18] have shown psychosocial stress to be an independent predictor of Type 2 diabetes and hyperglycemia. In addition, psychological stress is an independent predictor of the worsening of glycemic control in diabetic patients [69] [Path C_1_].

Finally, the number of study participants in the diabetic range was severely limited in our sample. However, numerous studies of HRV have been done with Type 2 diabetes and various other patient groups, with suggestion that more studies on healthy populations (such as the present sample) were needed [70]. Adding the self-reported diagnosis of hypertension and hyperglycemia as binary indicators to the mediation models did not change the reported pattern of results or our conclusions. Participants were volunteers; selection bias may have occurred between participating and non-participating employees such that non-participating employees may have been unhealthier and less willing to reveal personal medical data as compared to healthier participants who were more willing to attend a free physical examination. Our sample appears to be apparently healthy as only about 12% indicated a diagnosis of hypertension and about 2% indicated a diagnosis of hyperglycemia. When these diagnoses were added to the structural equation models the results, and therefore our conclusions, remained unchanged. However, if our sample is biased our estimates are likely to underestimate the associations of work stress and glycemic status as well as the mediating role of ANS measures. Despite these limitations the rather large healthy sample in the present study allows some confidence in the validity of the reported mediating effect.

### Conclusion

New strategies are necessary to counteract the high, and still increasing, prevalence of adverse glycemic status that may result in pre-diabetes and Type 2 diabetes. Therefore, the identification of protective factors, such as decreasing the Effort-Reward-Imbalance ratio, are important building blocks in the development of primary prevention strategies. On the basis of our data, we conclude that work stress may be an additional factor promoting hyperglycemic states. If supported by prospective evidence, these results point to a potential new avenue for the primary prevention of glycemic dysregulation.

## Appendix A

$$\text{Path A}1\;\left[\text{M}1\;=\;B_{0}+\;B_{1}\text{X }+\;B_{2}{\text{C}}_{1}\;+\;B_{3}{\text{C}}_{2}+\;B_{4}{\text{C}}_{3}+\;e\right]$$ (A.1)

$$\text{Path A}2\;\left[\text{M}2\;=\;B_{0}+\;B_{1}\text{X}+\;B_{2}{\text{C}}_{1}+\;B_{3}{\text{C}}_{2}+\;B_{4}{\text{C}}_{3}+\;e\right]\;$$ (A.2)

$$\text{Path B}1\;\left[\text{Y }=\;B_{0}+\;B_{1}{\text{M}}_{1}\;+\;B_{2}{\text{C}}_{1}+\;B_{3}{\text{C}}_{2}+\;B_{4}{\text{C}}_{3}+\;e\right]$$ (A.3)

$$\text{Path B}2\;\left[\text{Y }=\;B_{0}+\;B_{1}{\text{M}}_{2}+\;B_{2}{\text{C}}_{1}+\;B_{3}{\text{C}}_{2}+\;B_{4}{\text{C}}_{3}+\;e\right]$$ (A.4)

$$\text{Path C }\left[\text{Y }=\;B_{0}+\;B_{1}\text{X}+\;B_{2}{\text{C}}_{1}+\;B_{3}{\text{C}}_{2}+\;B_{4}{\text{C}}_{3}+\;e\right]$$ (A.5)

$$\text{Path C’ }\left[\text{Y }=\;B_{0}+\;B_{1}\text{X}+\;B_{2}M_{1}\;+{\text{ B}}_{3}{\text{M}}_{2}\;+{\text{ B}}_{4}{\text{C}}_{1}\;+{\text{ B}}_{5}{\text{C}}_{2}\;+{\text{ B}}_{6}{\text{C}}_{3}\;+\;e\right]$$ (A.6)

Proportion mediated = (Indirect effect / total effect) (A.7)

**Applied STATA command (version 12.1 MP update level 23March2013):**

#*delimit*;

*sem*(*log*(*RMSSD*) *←ERI AGE SEX*)

(*hba*1*c ←log*(*RMSSD*) *ERI AGE SEX*)

*method*(*mlmv*) *vce*(*bootstrap*, *strata*(*STUDY SITE*) *reps*(5000) *seed*(12345));

#*delimit cr*

**Postestimation using:**

estat teffects

## Abbreviations

C: Covariate
ERI: Effort-Reward Imbalance
HRV: Heart rate variability
MICS: Mannheim Industrial Cohort Study
M: Mediator
vmHRV: vagally-mediated HRV
X: Independent Variable
Y: Dependent Variable
